# Transcriptome analysis reveals the crucial function of hyperoside in inhibiting anthocyanin accumulation in grape (*Vitis vinifera* L.) fruits by inducing *VvMYB62*

**DOI:** 10.3389/fpls.2023.1119749

**Published:** 2023-03-07

**Authors:** Ling Su, Man Zhang, Yudie Zhang, Yingchun Chen, Liying Yang, Yongmei Wang, Yangbo Song, Lei Gong

**Affiliations:** ^1^ Shandong Academy of Grape, Shandong Academy of Agricultural Sciences, Jinan, China; ^2^ College of Horticulture Science and Technology, Hebei Normal University of Science and Technology, Hebei, China; ^3^ Hebei Key Laboratory of Horticultural Germplasm Excavation and Innovative Utilization, Qinhuangdao, Hebei, China; ^4^ College of Agriculture and Animal Husbandry, Qinghai University, Xining, China

**Keywords:** anthocyanin, grape, hyperoside, transcriptome, veraison

## Abstract

**Introduction:**

The formation of color in plants is significantly dependent on anthocyaninpigments. Grape species vary in color due to the differences in anthocyanin accumulation. It is widely recognized that both biotic and abiotic conditions may have an impact on anthocyanin synthesis in plants. The underlying molecular mechanisms by which external application of hyperoside impacts anthocyanin formation in grapes, however, have received little attention.

**Methods:**

In the current study,the transcriptome of Gemstone seedless grape was examined using high-throughput RNA sequencing at various developmental stages reply to both control and hyperoside treatments.

**Results:**

The results of this study suggested that the major genes controlling anthocyanin accumulation in response to the externalinjection of hyperoside could be VvMYB62, VvPAL, VvCHS, and VvF3’5’H.Quantitative reverse transcription PCR (RT-qPCR) results were used to confirm the changes in the expression levels of the genes encoding the anthocyanin biosynthesis pathway under the control and hyperoside treatments. Using a transient transformation system, it was discovered that VvMYB62 was shown to regulate the anthocyanin accumulation at both the transcriptional and posttranslational levels and could be influenced by the external administration of hyperoside. In grape embryogenic calli, hyperoside could specifically suppress theexpression of VvMYB62 and anthocyanin accumulation. In this instance, the VvMYB62 characterisation brought attention to the significance of exogenous hyperoside-induced anthocyanin accumulation. Therefore, the results demonstrated that VvMYB62 could be hindered in the process of grape during anthocyanin accumulation caused by hyperoside.

**Discussion:**

These findings offer excellent candidate genes in the future breeding of novel grape varieties in addition to serving as a crucial reference for understanding the underlying molecular processes of hyperoside suppression of anthocyanin formation in plants.

## Introduction

One of the oldest fruit woody vine species in the world is the grape (*Vitis vinifera* L.), which is a member of the grape family or the Vitaceae (Galet, 1988). Color change is one of the most obvious indicators of fruit ripening and fruit color in table grapes is a critical indicator of the market value. Alternatively, poor fruit coloring in the mature stages has a major impact on the economic worth of grapes.

The Gemstone seedless grape is a popular variety that has crisp, seedless meat, and brilliant red-purple and thin skin. The purplish-red color of this fruit is closely linked to its anthocyanin accumulation. Anthocyanin is a water-soluble natural pigment that is prevalent in plants (Khoo et al., 2017). The amount of anthocyanin changes at the same time as the fruit matures. The process of accumulation of anthocyanin has been thoroughly investigated. Anthocyanin is part of the phenolic group (Wallace and Giusti, 2015), have powerful antioxidant effects, and have shown a unique role in health. Pelargonidin, cyanidin, delphinidin, peonidin, and malvidin are the anthocyanidins that are most often included (Tanaka et al., 2008).

Anthocyanin is produced through phenylpropanoid metabolic pathways and flavonoid biosynthesis and are also regulated by multiple enzymes including cinnamate-4-hydroxylase (*C4H*) and coumaric acid COA ligase (*4CL*) for 4-coumaryl COA formation (Kim et al., 2007). Next, 4-coumaryl COA is catalyzed by chalcone synthase (*CHS*) to produce yellow chalcone, which is subsequently catalyzed by chalcone isomerase (*CHI*) and flavanone 3-hydroxylase (*F3H*) to form dihydroflavanol. Dihydroflavanol is catalyzed with 3’-hydroxylase flavonoid (*F3’H*) and 3’flavonoid. Dihydroquercetin and dihydromyricetin, precursors of anthocyanin production, are formed under the catalysis of 5’-hydroxylase (*F3’5’H*). Colorless anthocyanin is formed by dihydroflavanol-4-redox (*DFR*) and catalysis dioxygenase/anthocyanin synthetase (*LDOX/ANS*) (Saito et al., 2013; Wang et al., 2017; Chen et al., 2018; Peng et al., 2019; ). Finally, anthocyanin form glycosidic bonds under the effect of glucosyltransferase and are subsequently converted into stable anthocyanin (Yang et al., 2018).

Hyperoside is a flavonol glycoside compound and a potent monomer extract of *Abelmoschus* sp. (mallow or the Malvaceae family) (Raza et al., 2017). Hyperoside is an organic compound that has certain antioxidant effects (Wang et al., 2022). For example, hyperoside isolated from methanol extract from nut leaves and tested for its antioxidant potential was found to have a peroxide-free radical recovery activity. According to studies, the flavonoid hyperoside may function as a signal to extend the time that okra plants are in flower (Yang et al., 2020). Additionally, hyperoside might extend the time needed for optimal okra pollination and encourage the development of pollen tubes in the style (Dong et al., 2021). Hyperoside administration could also speed up seed germination, enhance fruit size, and encourage the formation of flavonoids in okra seeds (Yang et al., 2021). We learned that the current mechanism is: hyperoside further affects flavonoid biosynthesis through CDPK6-MYB30-UFGT (Yang et al., 2020). However, the impact of hyperoside on plant anthocyanin is unknown at this time.

This study on the effect of hyperoside treatment on anthocyanin provides the groundwork for future research on the underlying mechanisms of grape fruit discoloration. In this study, we treated the Gemstone seedless grape with hyperoside, combined with transcriptome sequencing and RT-qPCR validation results, six candidate genes were screened out. In addition, the effects of the hyperoside treatment on the accumulation of anthocyanin were investigated *via* grape callus and fruit transformation.

## Materials and methods

### Plant species, experimental conditions, and treatment

Gemstone seedless viticulture at Shandong Academy of Grape, and each treatment selected 1 vine with the same growth potential, total 6 vines. Each vine was sprayed with 50mg/L hyperoside or treated with the buffer as a control. The process was sprayed twice, first on June 21^st^ and second on July 9^th^. The spraying site was more than 2 lines of all leaves. Hyperoside reagent and the buffer was used to spray Gemstone seedless grapes from the early fruit stage to the ripening stage. The sample processing period was split into four stages (20 days at each stage for a total of 80 days) as follows: July 16 (B2, pre-color inversion stage), August 6 (B3, color reversal stage), August 26 (B4, early maturation stage), and September 16 (B5, complete ripening stage). Three copies of the experiment were carried out and the treated plants were immediately used for tissue sampling. Three fruits from the same plant were used as a treatment and each treatment sample was stored at -80°C until it was used.

### Determination of anthocyanin

Anthocyanin was extracted using the previous report (Fang et al., 2019), with minor modifications. Each sample and each repeat of the different treatments and different periods were powdered in liquid nitrogen. Afterwards, 10 ml methanol: acetic acid (99:1) solution was added with the ground-up sample completely immersed in the solution (pump if needed), blocked, and the sample was extracted in darkness for 24 hours (shaking could be repeated). 1 ml of the supernatant from the combination was combined with 4 ml of KCl buffer (pH=1.0) after the mixture was centrifuged for 10 minutes at 8,228 g. The measurements of OD levels were taken at 530 and 700 nm. Afterwards, 1 ml of the aforementioned extract was then mixed with 4 ml of a NaAc buffer (pH=4.5) and the mixture was incubated for 15 minutes at ambient temperature. The anthocyanin content was calculated as follows: C=ΔA ×5×0.005×1000×449.2/(26900×0.5); ΔA = (A530-A700) (pH = 1.0) - (A530-A700) (pH = 4.5). Where C is the anthocyanin content and A530 and A700 are the optical density values of sample solutions. Triplicates of each biological sample were created.

### Total RNA extraction, construction, and sequencing of the cDNA library

To assure the use of certified samples for transcription sequencing, the purity, concentration, and integrity of RNA samples are detected using cutting-edge molecular biology equipment. After the detection of qualified samples, the cDNA library was constructed as follows: (1) Oligo magnetic beads (dT) were added to eukaryotic mRNA; (2) mRNA was randomly aborted using the fragmentation buffer; (3) using mRNA as a model, the first and second strands of cDNA were then synthesized sequentially; and (4) the purified bacterial cDNA was repaired at the end, the A-tails were added, and the DNA was sequenced. (5) PCR enrichment was used to create the cDNA library. The library was built, and the RT-qPCR technique was utilized to precisely measure the real concentration of the library (> 2 nM) in order to ensure quality. Following a qualified inspection of the database, different libraries shall conduct pooling based on the target off-line data quantity. The sequencing was taken using the BMKCloud (https://www.biocloud.net).

### GO and KEGG enrichment analysis

The Gene Ontology Consortium created the Gene Ontology(GO) database in 2000 as a structured, standardized biomonitoring system (Ashburner et al., 2000). It intends to generate a standardized knowledge vocabulary on genes and their products, which applies to various species. Based on the non-central hypergeometric distribution of Wallenius, GOseq R software packages carried out GO enrichment analysis of differentially expressed genes (DEGs).

Different genetic products work together to carry out biological processes in organisms, and pathway annotation study of DEGs may assist in better understanding the role of the genes. KEGG (Kyoto Encyclopedia of Genes and Genomes) (Kanehisa et al., 2004) is a database for systematically analyzing gene function and genomic information, making it easier to better understand genomes. The information about genes and expression is usually investigated as a complete network. An enrichment analysis of the significance of pathways was performed to determine those pathways that were significantly enriched in DEGs in relation to the overall genome background using a hypergeometric test based on Pathway in the KEGG database. The statistical enrichment of DEGs was evaluated using the KOBAS program in the paths of KEGG (Bao et al., 2019). Methods with a reliable enrichment value (p-value < 0.05) were chosen for the analysis.

### RT-qPCR

For the RT-qPCR analysis, the total RNA of each sample was isolated from both treatments of the non-core gemstone. Each RNA sample’s residual DNA was extracted, and each cDNA sample was produced using Oligo dT in accordance with the manufacturer’s instructions (SuperScript III, Invitrogen, USA). The cDNA was diluted and used as a model. The gene-specific primers were made with Primer3 (https://primer3.org/). **Supplementary Table S2** presents the primer sequences. Quantitative fluorescence experiments were conducted with SuperReal PreMix (Probe) (Tianroot Biology, Beijing, China) and CFX was used (Bio-Rad, West Berkeley, California, USA) to assist in the analysis (Du et al., 2021). The delta CT methodology was used for the computation of gene expression results. All samples were split into three biological replicates of RT-qPCR.

### Putative gene selection

Flavonoid pathway-related DEGs were selected, such as *VvPAL* (phenylalanine lyase), *VvCHS* (Chalone synthase)*, VvF3’5’H* (Flavonoid 3,5—hydroxylase), and *VvMYB62* expressed during grape development. Using this website (http://www.bioinformatics.com.cn/), the heat map was produced.

### Cloning and sequencing of putative *VvMYB62*

Particular primers were employed to isolate the presumed *VvMYB62* gene. The PCR fragment was purified and cloned in the pROKII cloning vector (Song et al., 2022). The vector and the PCR-amplified product were mixed and ligated, and processed into Escherichia coli (*E. coli*) DH5α competent cells in accordance with the manufacturer’s instructions. DNAMAN software was used to compare gene sequences with the selected gene sequences with transcriptome sequences.

### Analysis of transiently-transformed grape calli

The embryogenic callus of the grape was transformed in a transitory manner (Bao et al., 2017). The coding sequence (CDS) of the *VvMYB62* gene was reconstituted into a pROKII vector with a GFP marker sequence to build the recombinant plasmid 35S:VvMYB62-GFP. The recombinant plasmid was converted into *Agrobacterium tumefaciens* EHA105 cells. After thawing, 200 ul of the bacterial solution was absorbed by the pipette and placed in 5 ml of Yep, Rif, and Kanamycin solution at 180 R/min on a shaker and 28°C for 20 hours, centrifuged at 5000 R/min for 10 minutes at room temperature. The supernatant was subsequently settled and the solution (4.44 g/L MS, 60 g/L sucrose, and 200 umol/L acetosyringone) was suspended again for two hours. The callus of a similar growth state (one month old) was chosen and the outer surface was transformed. The callus was submersed in the reassortment solution and sealed in a petri dish by means of using a vacuum pump for 20 minutes. The grape callus was cultured in the dark at 24°C for 48 hours on a solid medium (MS with 60 g/L sucrose, 7 g/L agar, and 200 umol/L acetosyringone). Grape callus transformed with an empty vector was used as control. In this experiment, the grape calli were pre-treated with a strain with overexpression *VvMYB62* and empty vector, then vacuumed, and then placed into a growing dish containing 50mg/L hyperoside solution for subsequent operations. One biological replicate was created from the grape calli treated in the same petri dish, and the analysis required at least three biological replicates. Calli were then collected, instantly frozen in liquid nitrogen, and then kept at -80°C for subsequent use.

### Instantaneous transformation of grape fruit

The fruits of Gemstone seedless grape were submerged in an *Agrobacterium* solution containing an empty vector and a vector for *VvMYB62* overexpression in accordance with the rapid transformation of the grape fruit technique (Yao, 2021). Grape fruits were placed in a box containing MS and sucrose during a photoperiod of 16 hours of light and 8 hours of darkness after overnight incubation at 26°C in the dark. After temporary metamorphosis, the grape epidermis’ color was seen. Anthocyanin concentration and gene expression levels were assessed between treatments.

### Statistical analysis

The error bars data were used to display the findings after each test was run in triplicate. Utilizing the GraphPad Prism 9 tool, the T-test approach was used to statistically analyze the data. P < 0.05 was represented by * and P < 0.001 by ***.

## Results

### Appearance and anthocyanin content of Gemstone seedless grape treated with hyperoside

**Figure 1:**
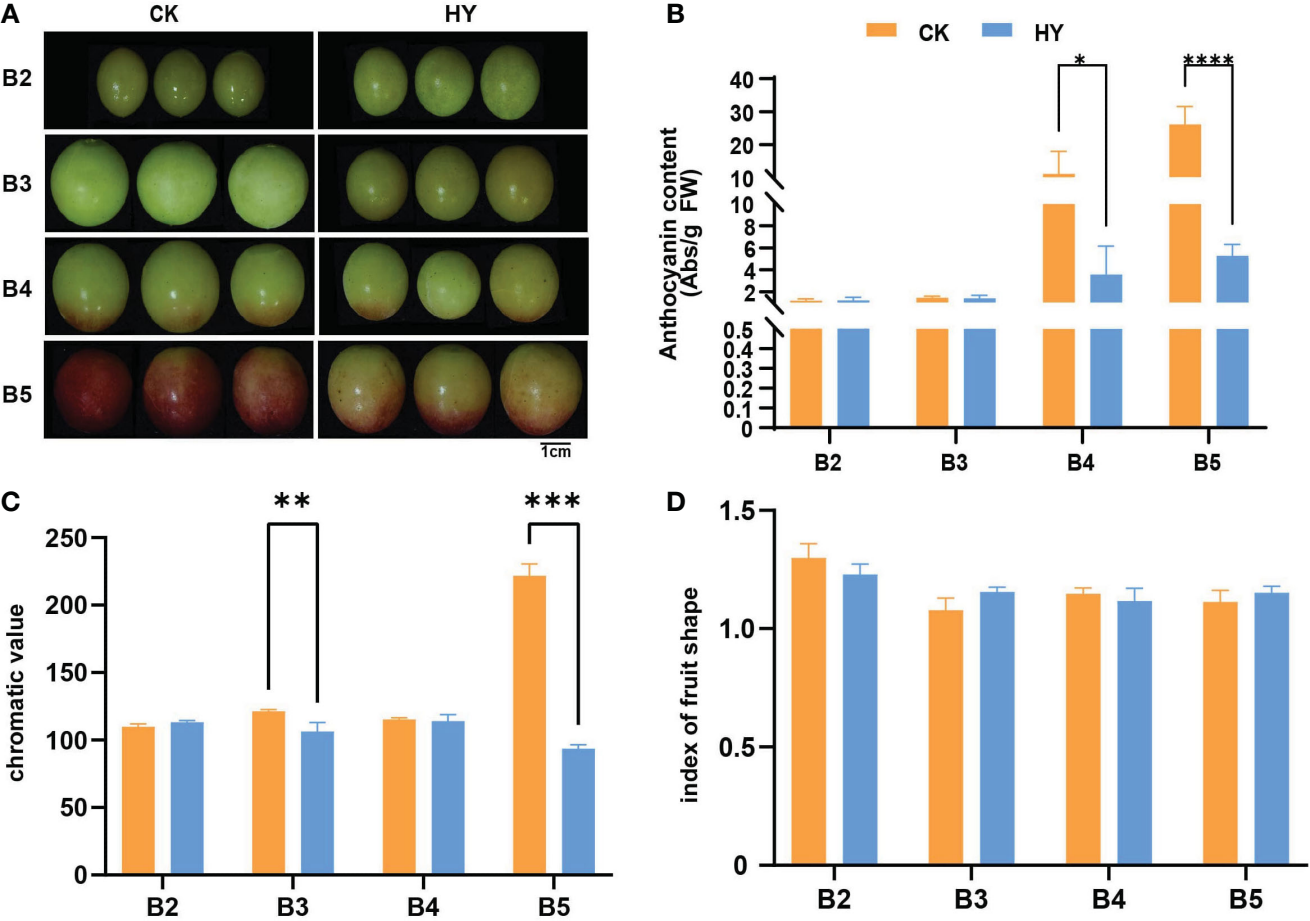
Phenotypic map of Gemstone seedless grape during fruit development when it was treated with the control and hyperoside(HY) treatments. **(A)** A representation of grape fruits in their young, late, and mature phases after being treated with hyperoside and under control. **(B)** The amount of anthocyanin in grape fruits at various growth stages and after various treatments. **(C)** The chroma value of grape fruits at various growth stages when subjected to various treatments. **(D)** The grape fruit form index at various growth stages under different treatments. The statistics reflect the mean ± SD of three biological replicates. Statistical significance: *P < 0.05; ***P < 0.01. **0.01<P<0.05, ****P<0.001.

To fully understand the effect of hyperoside treatment on anthocyanin content, we performed phenotypic analysis of grape fruits under two treatments.The changes in the color of grape fruit were observed at every stage.No obvious color differences were detected between the control and hyperoside-treated fruits at the color-turning and the early color-turning stages (stages B2 and B3). However, in the later stages of color alteration and fruit ripening (stages B4 and B5), the fruit color of hyperoside-treated samples was much paler than the control (**Figure 1A**). However, the fruit shape index did not significantly change from one period to the next (**Figure 1D**).

In view of the fact that treatment with hyperoside could significantly impact the antioxidant function of the fruit, the changes in anthocyanin content were further measured in both treatments. Phenotypic data showed that anthocyanin levels of both treatments increased substantilly at the B2-B5 stages with fruit development. Additionally, there were noticeable variations in anthocyanin content between both treatments at subsequent stages of color transfer and maturity (stages B4 and B5). Anthocyanin levels in hyperoside-treated fruit were significantly less than that of the control group (**Figures 1B, C**). These results suggest that the application of hyperoside could dramatically reduce the anthocyanin content in Gemstone seedless grapes. Transcriptome analysis was carried out on samples from the control and treatment groups at each of the five period groups in light of these phenotypic findings to identify the underlying molecular pathways of hyperoside-induced anthocyanin accumulation.

### Transcriptome sequencing, *de novo* assembly, and quality control analysis

Gemstone seed-free grape cDNA samples were sequenced with the Illumina sequencing platform. The selected clean reads were mapped using the grape reference genome (ftp://ftp.ensemblgenomes.org/pub/plants/release25/fasta/vitis_vinifera/). After a thorough analysis of the quality control data analysis, a total of 6.58 GB of clean data were obtained for each sample with a baseline Q30 percentage of 92.64% or more. Clean reads of each sample were compared sequentially with the reference genome of the grape and the effectiveness of the comparison varied from 83.91 to 94.87% (**Supplementary Table S1**). Based on the comparison results, DEGs were identified based on their levels of expression in various samples and functional annotations and enrichment analyses were conducted.

### Identification and screening of DEGs

**Figure 2:**
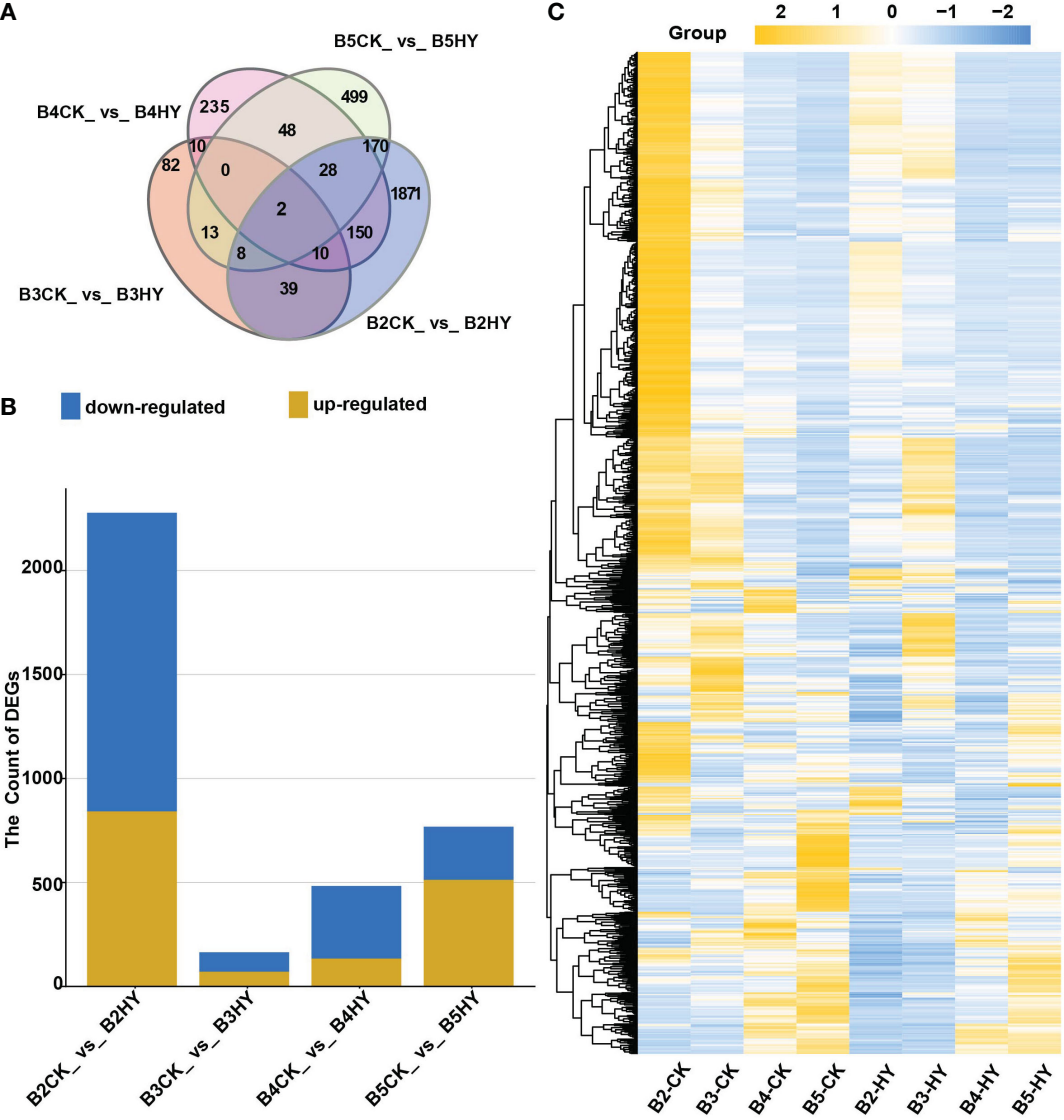
RNA sequence analysis was used to identify the number of differentially expressed genes (DEGs) in different treatment periods of Gemstone seedless grape. **(A)** Venn diagram representing the number of DEGs. **(B)** The overall total of DEGs used for each comparison. Yellow represents up-regulated and blue represents down-regulated. **(C)** The heat map analysis of DEGs.

The DEGs were screened between various treatments at the same developmental stage and explored the effects of hyperoside application on grape color transformation after the viability of the quality control data was confirmed. The following criteria were provided to see if the same gene is expressed differently in the two treatments: p-value<0.05 and the absolute value of Fold Change≥1.5. The DEGs were displayed using a Venn diagram (**Figure 2A**). Data revealed that there were 2,278 DEGs (two-fold up or down-regulated genes) in the B2CK_vs_B2HY group, of which 842 genes were up-regulated and 1,436 genes were down-regulated. There were 164 DEGs in the B3CK_vs_B3HY group, including 71 up-regulated and 93 down-regulated genes. In addition, 483 DEGs, including 134 up-regulated and 349 down-regulated genes, were found in the B4CK_vs_B4HY group. 513 up-regulated and 255 down-regulated genes showed differential expression at this stage compared to the previous phase (**Figure 2B**). Heat maps are used to depict DEG expression patterns in a more understandable way (**Figure 2C**).

### GO annotation analysis

**Figure 3:**
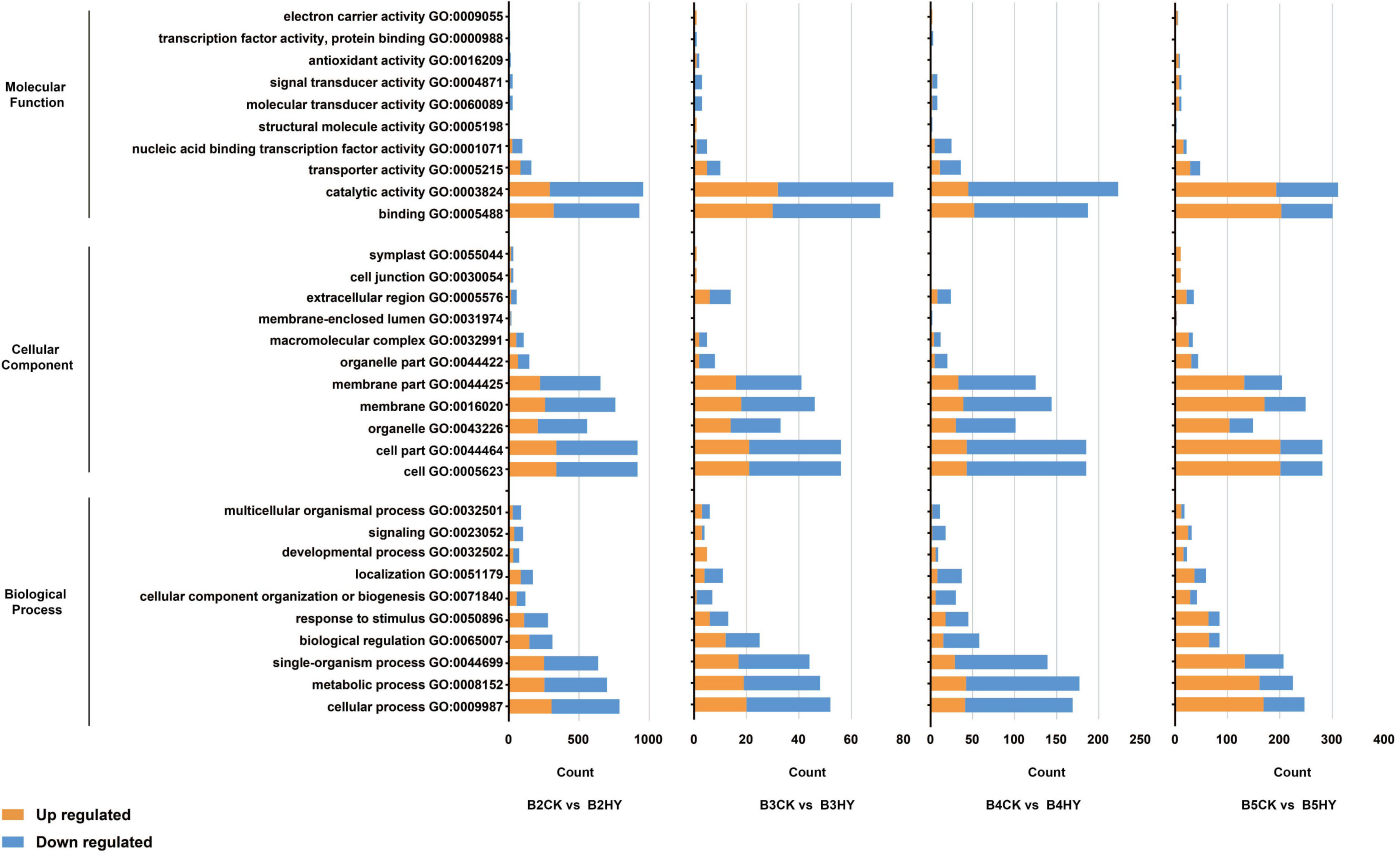
Comparison of hyperoside- and control-treated Gemstone seedless grapes at the B2, B3, B4, and B5 stages using GO enrichment analysis of differentially expressed genes (DEGs). The B2, B3, B4, and B5 stages stand for the early stage color transformation, the stage of color turning, the early maturity stage and the full maturity stage, respectively. Yellow represents up-regulated and blue represents down-regulated.

The three primary GO categories of “molecular function”, “biological processes”, and “cellular components” were applied to all DEGs (**Figure 3**). Four periods of GO enrichment analysis were completed. There were many terms in the categories “biological process,” “process,” “metabolism,” “the organization’s process,” “adjust,” “stimulus-response”, “the organization of cells or biological”, “localization”, “development”, as well as the “signal” and “process” of a multicellular organism. The term “extracellular area” was the most prevalent in the category “cell component”, which also included the terms “cell”, “cell part”, “organelle”, “membrane” and “organelle part”. The words “combination” and “catalytic activity”, “transport activity”, “nucleic acid combined with the activity of transcription factors”, “activists sensor”, “molecular sensor signal activity”, “cell”, “antioxidant activity” and “activity of transcription factors and protein activity” were some of the most frequently used ones in the category “molecular functions”.

### KEGG enrichment analysis

**Figure 4:**
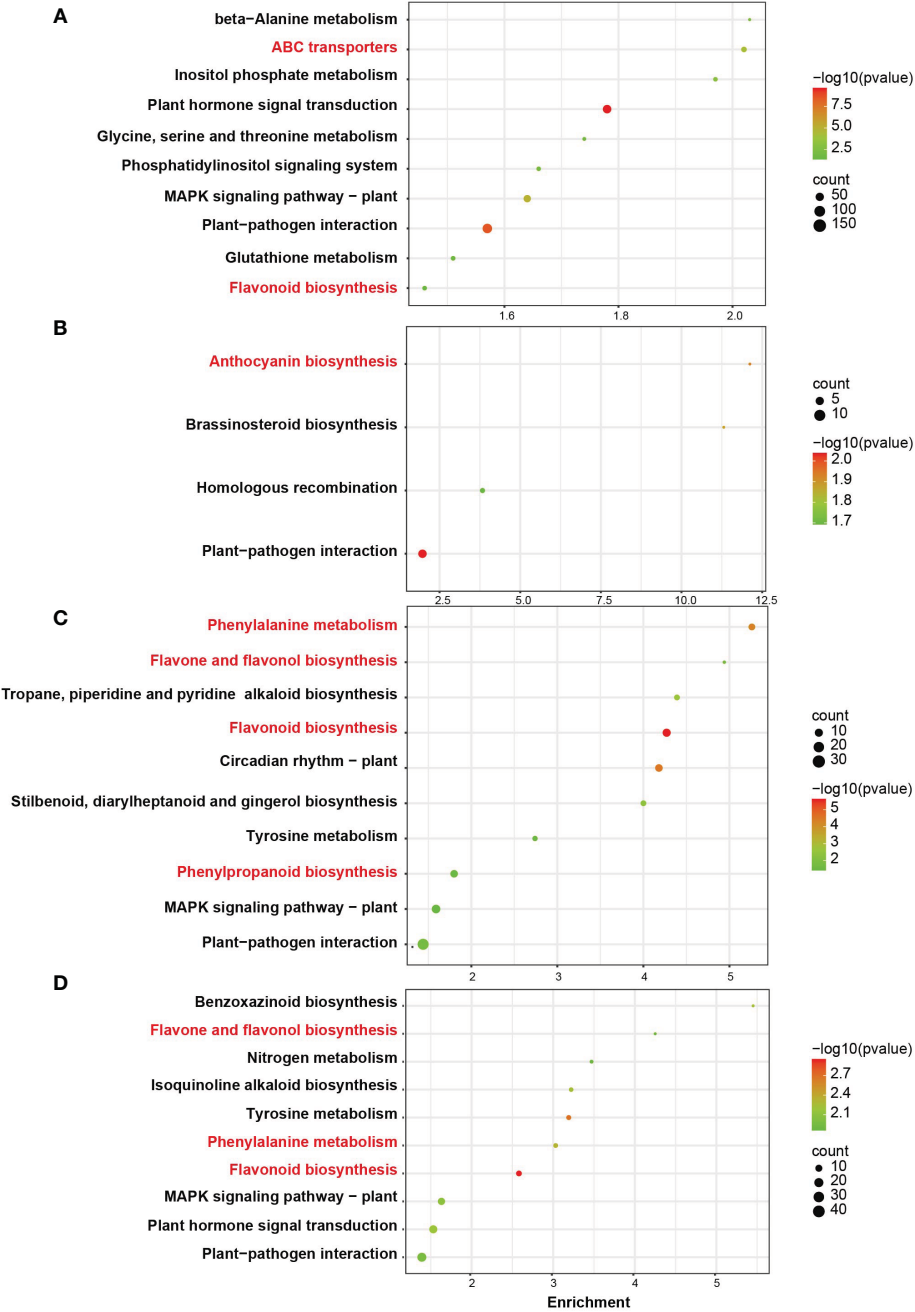
KEGG enrichment analysis of differentially expressed genes (DEGs) in Gemstone seedless grape that was treated with hyperoside and control at the B2, B3, B4, and B5 stages. The number of genes enriched in the pathway is represented by the size of the circle, and the color corresponds to the size’s p-value. Red labels are enriched in the top 10 sites, P < 0.05, and are associated with anthocyanin synthesis.

To further identify the biological functions of DEGs, a pathway analysis was performed using the KEGG database (**Figure 4**). The DEGs between B2_CK_vs_B2_HY groups were significantly enhanced in ten pathways. Both “ABC transporters” and “flavonoid biosynthesis” were closely linked to anthocyanin biosynthesis. DEGs between B3_CK_vs_B3_HY groups were significantly enhanced in four pathways, of which “anthocyanin biosynthesis” was the most relevant for this study. Four pathways in B4_CK_vs_B4_HY groups were linked to anthocyanin biosynthesis, including “phenylalanine metabolism”, “flavone and flavonol biosynthesis”, “flavonoid biosynthesis”, and “phenylpropanoid biosynthesis”.In the B5_CK_vs_B5_HY group, “flavone and flavonol biosynthesis”, “phenylalanine metabolism”, and “flavonoid biosynthesis” were correlated to anthocyaninbiosynthesis.

### Enzyme screening

**Figure 5:**
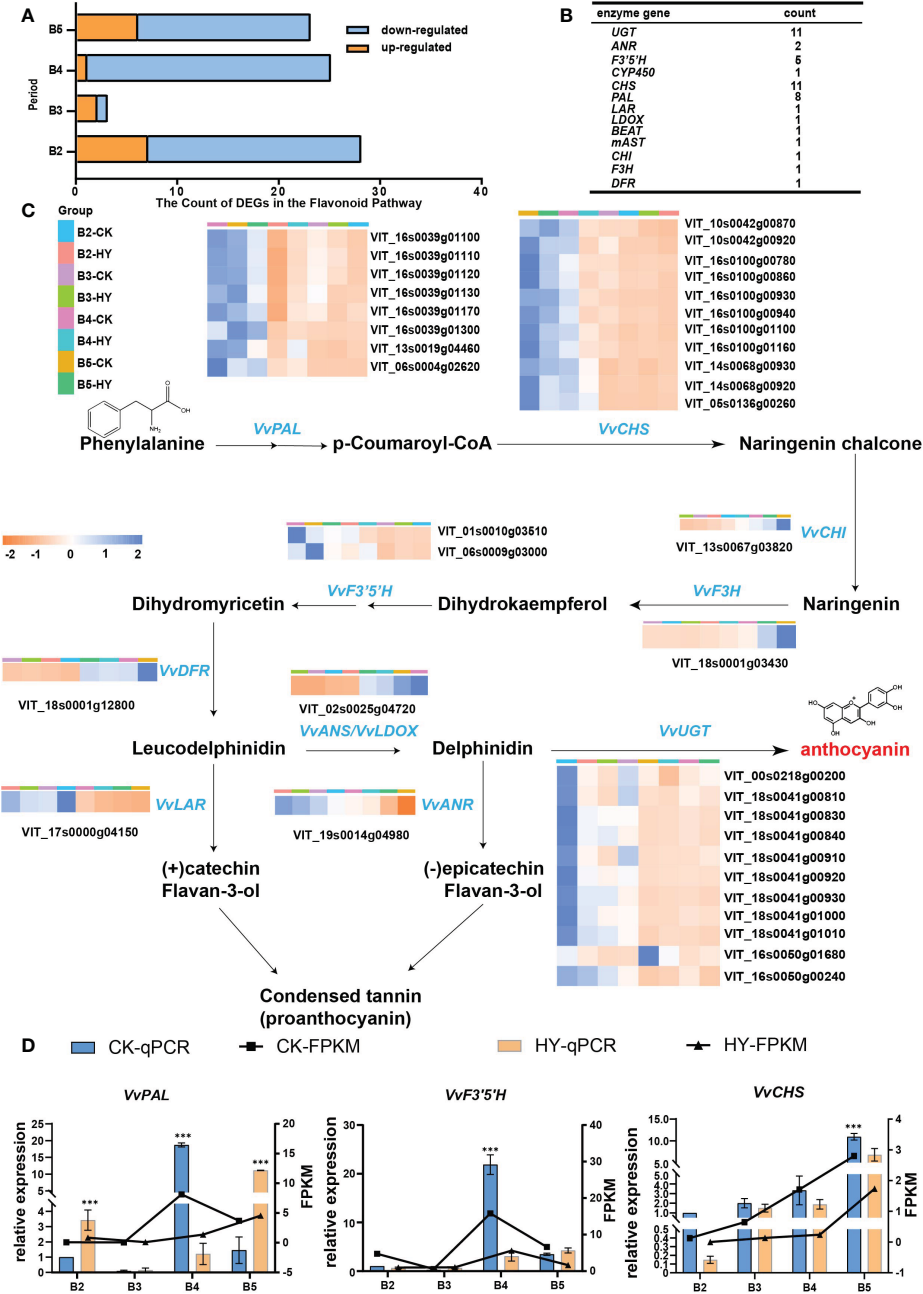
Screening and identification of enzymes. **(A)** Hyperoside-induced flavonoid metabolism pathway in Gemstone seedless grape fruit.Yellow represents up-regulated and blue represents down-regulated. **(B)** Data on the genes in the flavonoid biosynthesis pathway that were downregulated. **(C)** The pathway process in which the enzyme is located. Heat map illustrating changes in the anthocyanin metabolic pathways-related differentially expressed genes (DEGs) after treatment with hyperoside. **(D)** The gene expression levels of three enzymes (VvPAL, VvCHS, and VvF3’5’H) were determined by RT-qPCR. P< 0.05 and P< 0.01 are denoted, respectively, by the symbols * and ***.

The flavonoid biosynthesis pathway’s known genes were enumerated in the aforementioned four categories of comparison in order to better understand which genes are involved in the creation of color. Significantly more down-regulated genes were present than up-regulated ones. The B4 stage had the most DEGs that were down-regulated (**Figure 5A**). Statistics of the DEGs were presented in **Figure 5B**. The expression levels of the control and hyperoside treatments, as well as the B4 stage, were substantially different for a total of 22 DEGs involved in the synthesis of anthocyanin. The B5 stage has greater levels of gene expression than the B2 stage (**Figure 5C**).

In-depth explanations were provided for the metabolic pathway diagram used in this work. PAL catalyzes the formation of 4-coumaryl CoA from phenylalanine. Afterward, 4-coumaryl CoA is catalyzed by CHS to produce yellow chalcone, followed by CHI to form dihydroflavonol, and ultimately anthocyanin is synthesized. These three genes(*VvPAL, VvCHS*, and *VvF3’5’H*) with high expression were further examined using RT-qPCR verification. The outcomes demonstrated that the trend of fluorescence quantification-verified above gene expression was compatible with the transcriptome data, demonstrating the validity of the transcriptome data (**Figure 5D**).

### Transcription factor identification and screening

**Figure 6:**
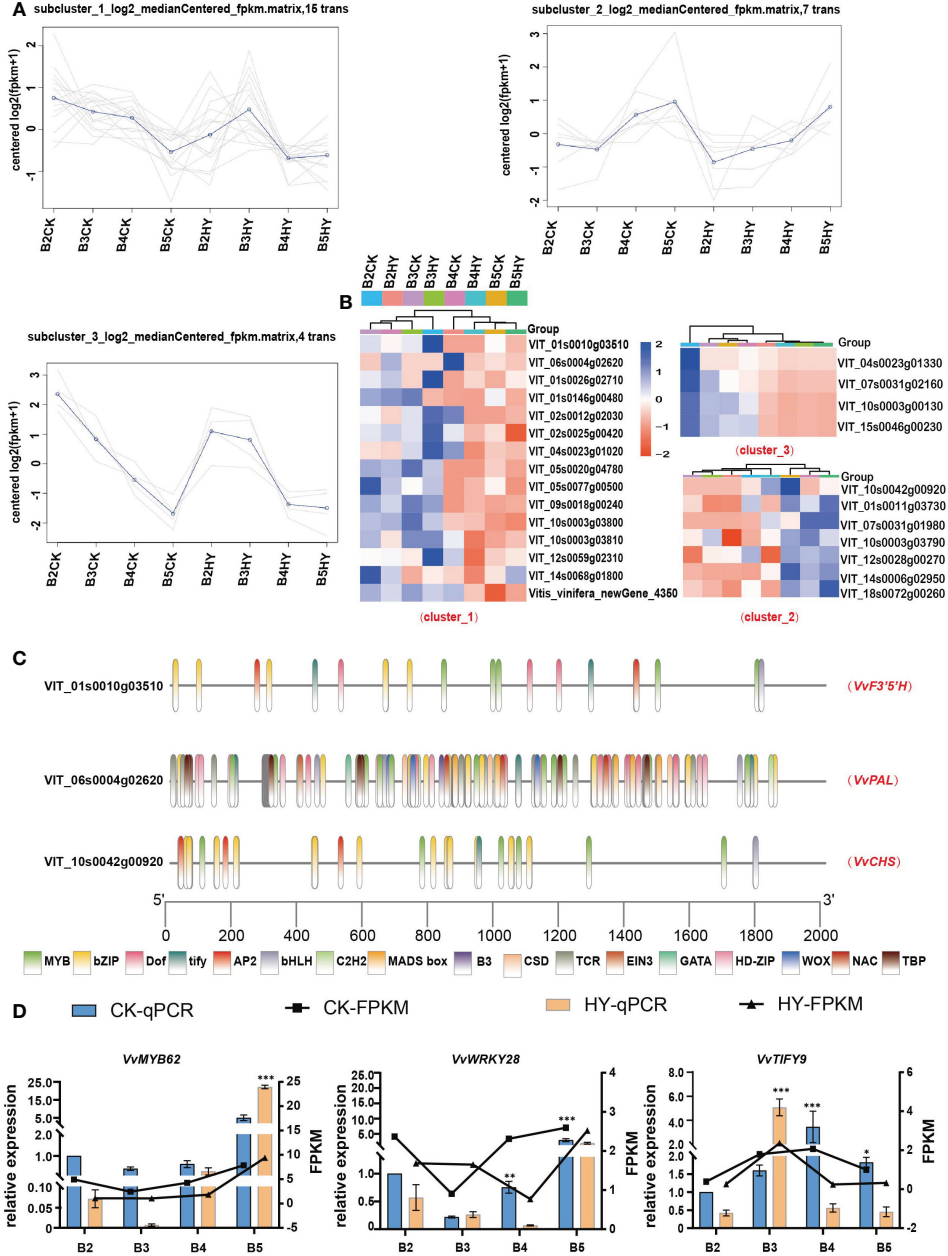
The screening and identification of transcription factors. **(A)** Co-expression trend analysis of transcription factors and enzymes. **(B)** Heat maps show the expression levels of selected transcription factors. **(C)** Analysis of promoter cis-acting elements. **(D)** RT-qPCR show the expression of transcription factors. *P<0.05, **0.05<P< 0.01, ***P< 0.01.

An investigation of the co-expression patterns of all DEGs and the three aforementioned enzymes was carried out in order to learn more about how the treatment with hyperoside affects the biosynthesis of anthocyanin. The analysis of the fourth phase DEGs and the co-expression of all transcription factors revealed a separation into three clusters (**Figure 6A**). According to the rule of growth and development, heat map research revealed that several transcription factors had the same expression pattern as the enzymes (**Figure 6B**). The promoter sites of the aforementioned three enzymes were found to be largely localized in the family of *MYB* transcription factors using promoter homeogenic elements (**Figure 6C**). Three transcriptionfactors,*VvMYB62,VvWRKY28*,and *VvTIFY9*,were chosen for further verification(**Figure 6D**). *VvMYB62* was chosen for overexpression investigation as a result.

### The influence of *VvMYB62* overexpression on grape callus

**Figure 7:**
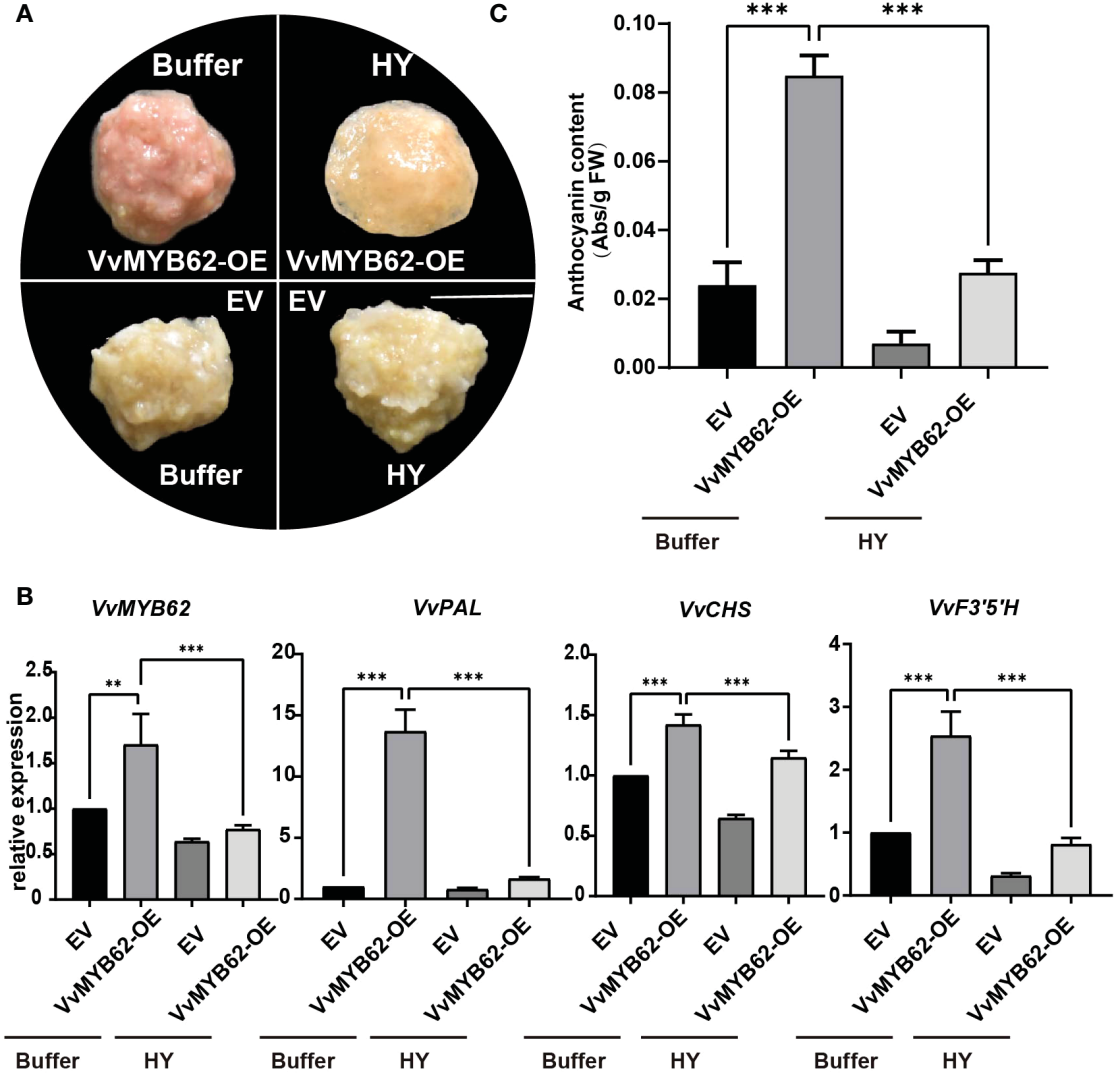
Exogenous hyperoside prevented grape calli from expressing *VvMYB62* and accumulating anthocyanin. **(A)** The appearance of the callus after four treatments. A 1 cm scale bar is used. **(B)** Levels of *VvMYB62*, *VvPAL, VvCHS*, and *VvF3’5’H* transcription. An internal control gene was *VvActin*. **(C)** The amount of anthocyanin in the CK and OE calli. The mean and standard deviation of three independent biological replicates are used to express values. Significant statistically: **0.05<P<0.01; ***P<0.01.

In plants, transient transformation is a reasonably quick method for detecting target gene expression (Sparkes et al., 2006). Grape calli were genetically altered using *Agrobacterium tumefaciens* to demonstrate that *VvMYB62* may respond to hyperoside to control anthocyanin accumulation. Phenotypic alterations were seen once the *VvMYB62* transcription factor was chosen for over-expression confirmation (**Figure 7A**). There were red callus lines where *VvMYB62* was overexpressed. Calli overexpressing *VvMYB62*, however, displayed reduced redness in a hyperoside-induced media. The control’s calli were white. The measurement of anthocyanin content (**Figure 7C**) and RT-qPCR analysis (**Figure 7B**) corroborated the alterations in anthocyanin in the callus. According to the spectrophotometric measurement, VvMYB62-OE increased anthocyanin accumulation above the control. However, the capacity of VvMYB62-OE to promote anthocyanin accumulation was diminished after exogenous hyperoside therapy. RT-qPCR was used to assess the transcriptional levels of the genes involved in the anthocyanin biosynthesis pathway. According to these findings, *VvMYB62, VvPAL, VvCHS*, and *VvF3’ 5’H* were all up-regulated to varying degrees in VvMYB62-OE, while they were all down-regulated in VvMYB62-OE was hyperoside-mediated.

### Influence of *VvMYB62* overexpression on grape fruits

**Figure 8:**
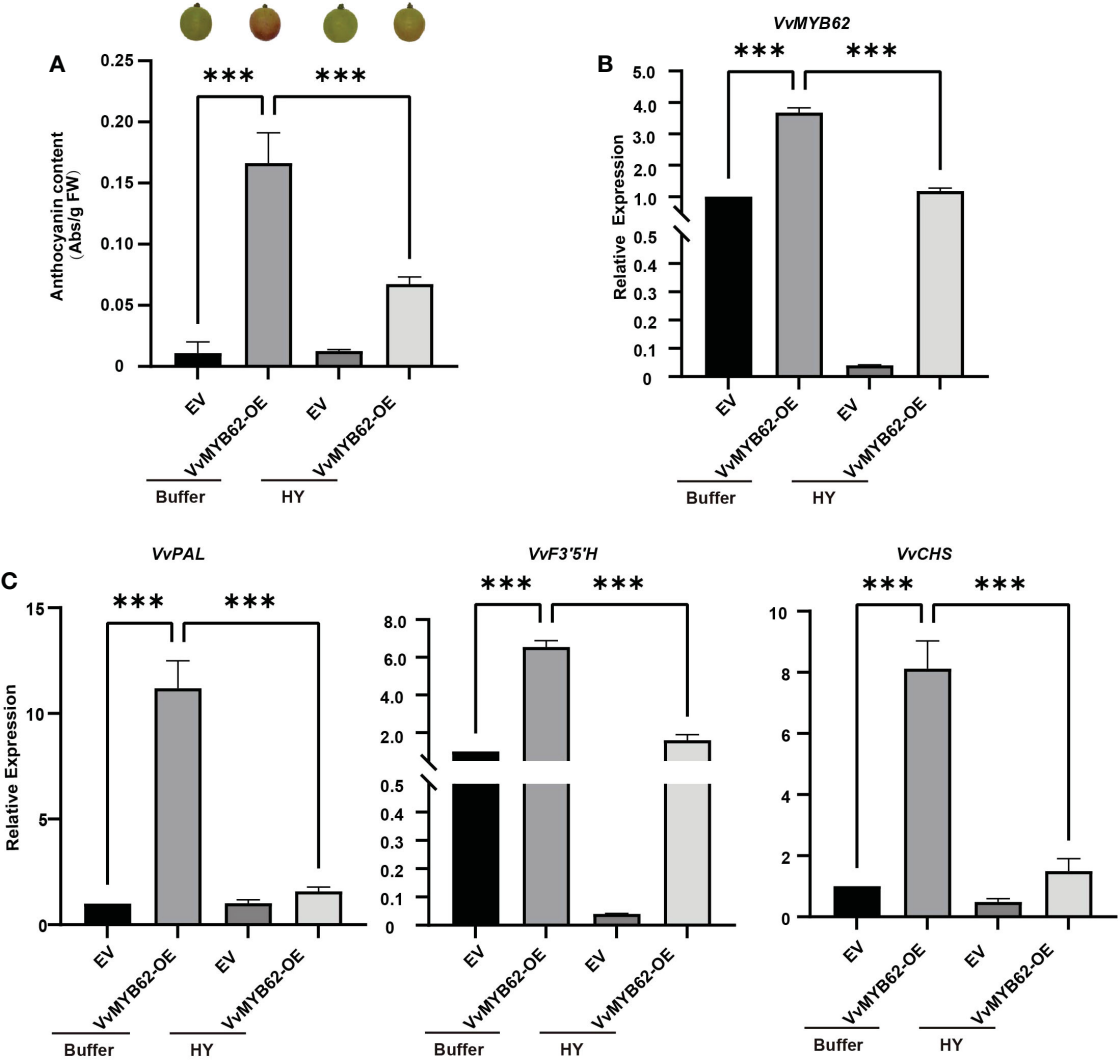
The instantaneous transformation of grape fruits. **(A)** The anthocyanin concentration and phenotypic diagram of grapes following transient overexpression. **(B)** RT-qPCR was used to identify the relative expression of *VvMYB62* in the four treatments after transformation. **(C)** RT-qPCR was used to identify the relative expression levels of *VvPAL, VvCHS*, and *VvF3’5’H* in the four treatments after transformation. The mean and standard deviation of three independent biological replicates are used to express values. Significant statistically: **0.05<P<0.01; ***P< 0.01.

The *VvMYB62* gene was transiently inserted into Gemstone seedless grape using Agrobacterium-mediated transfer in order to better investigate the function of the gene and its alterations after hyperoside treatment. Significant changes in fruit color between the treatment and control groups occurred after around 4 days of cultivation (**Figure 8A**). The trend difference of *VvMYB62, VvPAL, VvCHS*, and *VvF3’5’H* between the control and treatment groups was further established by RT-qPCR (**Figures 8B, C**). This study also showed that hyperoside treatment may minimize the coloring impact while *VvMYB62* might increase the coloring of grape fruits.

## Discussion

The grape,a type of berry, is typically spherical or oval (Kupe, 2020). Grapes come in six different color varieties around the world: white, green, yellow, red, black, and purple (Walker et al., 2007). Because of their vibrant color, delectable flavor, and great nutritious content, customers adore grapes (Swami et al., 2014).

The underlying molecular mechanisms of anthocyanin biosynthesis induced by hyperoside in Gemstone seedless grapes were revealed in this study using transcriptome analysis, and several important transcription factors and enzyme genes were identified. Further investigation into the processes underlying grape coloration is made possible by the clarification of the effects of hyperoside treatment on anthocyanin biosynthesis.

### Effects of hyperoside treatment on anthocyanin biosynthesis

It is generally recognized that both exogenous and internal variables, such as light, temperature, plant growth regulators, and nutritional status, have an impact on the production of flavonoids (Naik et al., 2022). This study showed a connection between endogenous anthocyanin accumulation and hyperoside administration externally. Hyperoside is a 3-O-galactoside of quercetin that encourages the flowering and seed formation of *Abelmoschus esculentus* (Naik et al., 2022). In order to monitor fruit growth, we sprayed each vine in this trial with 50mg/L hyperoside and buffer as a control. The findings show that Gemstone seedless grape anthocyanin content may be considerably reduced by exogenous hyperoside, delaying the stage of color change (**Figure 1**).

## Enzyme changes during anthocyanin biosynthesis

The first significant enzyme in the process for the manufacture of anthocyanin is *CHS*. And it has been identified and cloned from numerous plants (Whang et al., 2011). Changes in the expression of the *CHS* gene may have an impact on plant color. White or spotted flowers could result from petunia *CHS* gene overexpression (Wang et al., 2006). The second important enzyme in the pathway that produces anthocyanin is called *CHI* (Sun et al., 2019). One of the important enzymes in the initial stage of anthocyanin biosynthesis is called *F3H.* According to earlier research, strawberry fruits infected with *Agrobacterium* GV3101 carrying the F3H-RNAi gene expressed the *F3H* gene at a lower level than the control (Jiang et al., 2013). Additionally, there was a considerable decrease in the content of anthocyanin and flavonol. In this research, exogenous hyperoside administration decreased the expression of key enzymes in the anthocyanin biosynthesis pathway, including *VvPAL, VvAOMT3, VvCHS, VvCHI, VvF3H, and VvF3’5’H* (**Figure 5**). Hyperoside may decrease anthocyanin production in grape grapes, as evidenced by the expression of associated enzymes throughout this process.

## Changes in transcription factors during anthocyanin biosynthesis

The *TIFY* gene family is a significant transcription gene family in the plant world. It was formerly a member of the *ZIM* (zinc finger protein expressed in inflorescence meristem) gene family (Vanholme et al., 2007). The spatiotemporal expression pattern suggests a potential link between the *PpTIFY* gene and anthocyanin accumulation (Ma et al., 2018). And whereas most *PpTIFY* genes dramatically decreased in expression during fruit ripening, the amount of *PpTIFY* gene expression was highest in young leaves. Anthocyanin have been found to accumulate significantly in plants that overexpress the *MYB62* gene (Devaiah et al., 2009).*LOB* domain (*LBD*) proteins are classified as conserved transverse organ boundary (*LOB*) proteins that are plant-specific transcription factors. They are mostly found in *LBD* proteins, which in general control the synthesis of anthocyanin and the nitrogen reaction. Previous research has discovered that *WRKY28* and anthocyanin share a common ancestor (Zhang et al., 2020). In this investigation, we discovered that several transcription factors, including *VvMYB62, VvTIFY9, VvTIFY5A, VvWRKY28, VvLOB41*, and *VvERF110*, displayed a significant down-regulation trend during the B2-B5 stages after treatment with hyperoside (**Figure 6**). Therefore, it was proposed that hyperoside treatment would alter the down-regulation of these transcription factors, so reducing anthocyanin formation and delaying the stage of grape coloration.

Experiments on transient expression were done on the grape fruits and the embryogenic callus. Furthermore, it was discovered that following the overexpression of *VvMYB62*, the anthocyanin content and target gene expression greatly increased (**Figures 7**, **8**). As a result, *VvMYB62* could be able to favorably influence anthocyanin synthesis. In this study, hyperoside treatment in Gemstone seedless grape decreased anthocyanin concentration and downregulated target genes. These findings further indicated that grape hyperoside treatment-induced anthocyanin production could be adversely regulated by *VvMYB62*.

**Figure 9:**
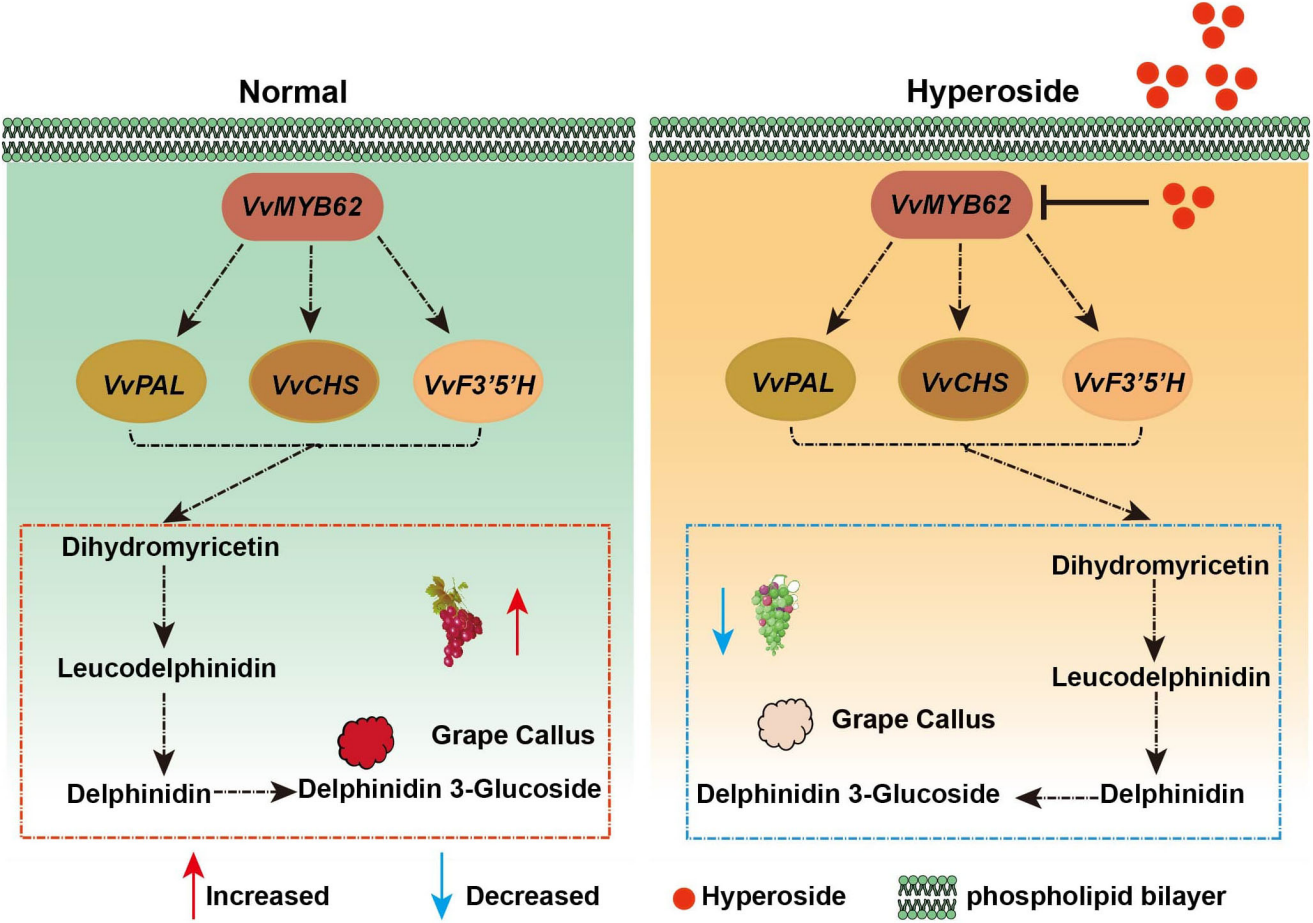
The metabolic routes for anthocyanin in grape fruits in response to hyperoside treatment are depicted in the working model. We put forth a molecular method that would prevent the production of anthocyanin by boosting *VvMYB62* expression by spraying hyperoside on grape fruit. The expression level of *VvMYB62* might be elevated and anthocyanin accumulation could be further encouraged in the absence of hyperoside. However, when grape fruits were given further hyperoside treatment, *VvMYB62* expression was repressed and anthocyanin accumulation was constrained.

Based on the outcomes of this experiment, a biological model was created to clarify the part that hyperoside plays in the accumulation of anthocyanin (**Figure 9**). According to the research, the flavonol compound hyperoside prevents the accumulation of anthocyanin in grape fruits by lowering the level of transcription factor VvMYB62’s expression, which in turn affects the expression of related enzyme genes in the flavonoid pathway.These extensive data sets offer the groundwork for further research into the function of hyperoside therapy in anthocyanin formation as well as the underlying processes of grape hue.

## Conclusion

The fundamental molecular mechanism of hyperoside-inducing anthocyanin production in Gemstone seedless grape was clarified in this work using transcriptome sequencing. Seven transcription factors and 22 differentially expressed enzymes were found; they were then categorized into three groups. These findings suggested that hyperoside treatment could prevent anthocyanin from changing color. The impact of hyperoside on the biosynthesis of anthocyanin is now well-understood thanks to this work.

## Data availability statement

The original transcriptome sequencing data involved in this paper has been uploaded to the NCBI with the upload number PRJNA935423.

## Author contributions

LG contributed to the conception and design of the study; MZ, YZ organized the database; LY, YW and YC performed the statistical analysis; LS wrote the manuscript. All authors contributed to the article and approved the submitted version.
