# Supplementary material for: Transcriptome analysis reveals the crucial function of hyperoside in inhibiting anthocyanin accumulation in grape (Vitis vinifera L.) fruits by inducing VvMYB62
Source: Front Plant Sci. 2023 Mar 7;14:1119749. doi: 10.3389/fpls.2023.1119749 (PMC10028066; doi:10.3389/fpls.2023.1119749)
Supplement: Supplementary file 2 [file Table_2.docx]

Supplementary Material

Transcriptome Analysis Reveals the Key Role of Hyperoside in Inhibiting Anthocyanin Accumulation in Grape (*Vitis vinifera* L.) Fruits by Inducing *VvMYB62*

LingSu^1^, ManZhang^2,3^, Yudie Zhang^2,3^, Yingchun Chen^1^, Liying Yang^1^, YongmeiWang^1^, LeiGong^1,*^

^1 Shandong Academy of Grape, Shandong Engineering Research Center for Grape Cultivation and Deep-processing, Key Laboratory of East China Urban Agriculture, Ministry of Agriculture and Rural Affairs, Jinan 250100, China.^

^2 College of Horticulture Science and Technology, Hebei Normal University of Science and Technology, Qinhuangdao 066004, Hebei, China^

^3 Hebei Key Laboratory of Horticultural Germplasm Excavation and Innovative Utilization^

*** Correspondence:**

Corresponding author: glflysky@163.com

# Supplementary Table S1 RNA-Seq data statistics of grapes

| **Samples** | **Clean reads** | **Clean bases** | **GC Content** | **%≥Q30** |
| --- | --- | --- | --- | --- |
| B-1-C1 | 21,675,782 | 6,486,222,006 | 46.36% | 95.68% |
| B-1-C2 | 20,572,099 | 6,159,143,542 | 46.03% | 94.81% |
| B-1-C3 | 20,858,683 | 6,241,405,470 | 46.04% | 95.20% |
| B-2-C1 | 33,086,879 | 9,892,274,198 | 44.92% | 92.64% |
| B-2-C2 | 20,370,712 | 6,098,174,694 | 46.08% | 95.54% |
| B-2-C3 | 26,138,102 | 7,821,596,736 | 46.30% | 95.76% |
| B-2-J1 | 20,360,075 | 6,093,455,456 | 46.50% | 95.14% |
| B-2-J2 | 19,464,396 | 5,826,176,064 | 46.26% | 95.55% |
| B-2-J3 | 19,815,400 | 5,932,013,280 | 46.22% | 95.47% |
| B-3-C1 | 21,855,294 | 6,545,280,948 | 46.74% | 95.63% |
| B-3-C2 | 20,909,710 | 6,259,470,782 | 46.02% | 94.87% |
| B-3-C3 | 20,089,622 | 6,013,822,522 | 46.21% | 94.49% |
| B-3-J1 | 20,342,259 | 6,090,315,306 | 46.26% | 94.48% |
| B-3-J2 | 20,829,720 | 6,234,305,206 | 46.41% | 94.64% |
| B-3-J3 | 23,221,314 | 6,950,790,518 | 46.16% | 94.79% |
| B-4-C1 | 27,664,705 | 8,276,397,718 | 46.44% | 94.23% |
| B-4-C2 | 20,245,625 | 6,058,777,330 | 46.44% | 93.92% |
| B-4-C3 | 19,408,486 | 5,809,144,382 | 46.23% | 94.73% |
| B-4-J1 | 20,337,868 | 6,083,852,908 | 47.19% | 93.51% |
| B-4-J2 | 27,931,094 | 8,359,258,214 | 47.14% | 94.76% |
| B-4-J3 | 28,427,076 | 8,501,079,982 | 47.60% | 95.04% |
| B-5-C1 | 24,031,739 | 7,190,988,918 | 47.33% | 95.18% |
| B-5-C2 | 25,926,441 | 7,758,056,874 | 46.67% | 94.73% |
| B-5-C3 | 24,225,372 | 7,248,023,710 | 46.61% | 95.14% |
| B-5-J1 | 21,776,263 | 6,513,593,614 | 48.42% | 95.07% |
| B-5-J2 | 21,383,572 | 6,399,824,376 | 46.94% | 95.23% |
| B-5-J3 | 22,763,057 | 6,810,476,512 | 47.73% | 95.10% |

# Supplementary Table S2 Primers for RT-qPCR analysis

| **Gene name** | **Forward prismer (5’ to 3’)** | **Reversre primer (5’ to 3’)** |
| --- | --- | --- |
| *β*-Actin | CTTGGCTGATAGGCTGGCGAAG | TCCACATCTGCTCAAAGGTGCTTAG |
| *VvPAL* | AGAATGGTGGCGGACTTCAG | TGTCGGTCCCTTGGTTCATG |
| *VvCHS* | CCCGGCCATTCTAGATGCAA | GTCAAACCTGGCCCAAAACC |
| *VvF3’5’H* | GACTTTGCGATGGTGGATGC | GATGGACTCTGCCTGCTCTC |
| *VvMYB62* | ACAGGCAAGGCAACTCAAGATCG | TTGGTAGTCACAGTATCAGCAGCAG |
| *VvTIFY9* | GGCACGAAGGAAGTCATTGC | CCGCGTATTGGATGTGAGGT |
| *VvWRKY28* | CATCCCTCCACGCCTAATTCCTTG | TCATCTCCATCTTCACACCCTCCTC |
